# Supplementary material for: Dietary magnesium supplementation improves lifespan in a mouse model of progeria
Source: EMBO Mol Med. 2020 Aug 16;12(10):e12423. doi: 10.15252/emmm.202012423 (PMC7539193; doi:10.15252/emmm.202012423)
Supplement: Supplementary file 1 — Appendix [file EMMM-12-e12423-s001.pdf]

# APPENDIX

## Table of contents


**Appendix Table S1**. The exact P values for Figure EV1
**Appendix Table S2**. The exact P values for Figure 1
**Appendix Table S3**. The exact P values for Figure EV2
**Appendix Table S4**. The exact P values for Figure 2
**Appendix Table S5**. The exact P values for Figure 3
**Appendix Table S6**. The exact P values for Figure 4
**Appendix Table S7**. The exact P values for Figure EV3
**Appendix Table S8**. The exact P values for Figure 5
**Appendix Table S9**. The exact P values for Figure EV4

**Appendix Table S1**. The exact P values for Figure EV1

| | **Figure EV1C** | **Figure EV1D** | **Figure EV1E** | **Figure EV1F** | **Figure EV1G** |
|---|---|---|---|---|---|
| wild-type versus untreated $Lmna^{G609G/+}$ | <0.0001 | <0.0001 | <0.0001 | <0.0001 | <0.0001 |
| wild-type versus treated $Lmna^{G609G/+}$ | 0.0154 | 0.0029 | 0.012 | 0.0278 | 0.0001 |
| untreated $Lmna^{G609G/+}$ versus treated $Lmna^{G609G/+}$ | 0.0199 | 0.0352 | 0.0096 | 0.0211 | 0.0001 |
| | One-way ANOVA and Tukey´s post hoc test | | | | |

**Appendix Table S2**. The exact P values for Figure 1

| | **Figure 1A** | **Figure 1B** | **Figure 1C** |
|---|---|---|---|
| wild-type versus untreated $Lmna^{G609G/+}$ | <0,0001 | <0,0001 | <0,0001 |
| wild-type versus treated $Lmna^{G609G/+}$ | 0,0105 | 0,006 | 0,0296 |
| untreated $Lmna^{G609G/+}$ versus treated $Lmna^{G609G/+}$ | 0,0041 | 0,0102 | 0,0074 |
| | One-way ANOVA and Tukey´s post hoc test | | |

**Appendix Table S3**. The exact P values for Figure EV2

| | **Figure EV2A** | **Figure EV2B** | | **Figure EV2C** | **Figure EV2E** |
|---|---|---|---|---|---|
| wild-type versus untreated Lmna$^{G609G/+}$ | <0.0001 | 0.0001 | <0.0001 | <0.0001 | <0.0001 |
| wild-type versus treated Lmna$^{G609G/+}$ | 0.0052 | 0.1493 | 0.002 | 0.0607 | <0.0001 |
| untreated Lmna$^{G609G/+}$ versus treated Lmna$^{G609G/+}$ | <0.0001 | 0.0259 | 0.0004 | 0.001 | 0.9903 |
| | One-way ANOVA and Tukey´s post hoc test | | | | |

| | **Figure EV2D** | | | **Figure EV2F** | |
|---|---|---|---|---|---|
| wild-type versus untreated Lmna$^{G609G/+}$ | <0.0001 | <0.0001 | <0.0001 | <0.0001 | 0.7051 |
| wild-type versus treated Lmna$^{G609G/+}$ | <0.0001 | <0.0001 | 0.0389 | 0.0215 | 0.7879 |
| untreated Lmna$^{G609G/+}$ versus treated Lmna$^{G609G/+}$ | 0.5409 | <0.0001 | 0.0376 | 0.0372 | 0.9892 |
| | One-way ANOVA and Tukey´s post hoc test | | | | |

**Appendix Table S4**. The exact P values for Figure 2

| | **Figure 2A** | **Figure 2B** | **Figure 2C** | **Figure 2D** | **Figure 2E** |
|---|---|---|---|---|---|
| wild-type versus untreated $Lmna^{G609G/+}$ | <0.0001 | <0.0001 | <0.0001 | <0.0001 | 0.0023 |
| wild-type versus treated $Lmna^{G609G/+}$ | <0.0001 | 0.0006 | <0.0001 | 0.1232 | 0.502 |
| untreated $Lmna^{G609G/+}$ versus treated $Lmna^{G609G/+}$ | 0.0292 | 0.0273 | 0.011 | 0.017 | 0.0407 |
| | One-way ANOVA and Tukey´s post hoc test | | | | |

**Appendix Table S5**. The exact P values for Figure 3

| | **Figure 3C** | **Figure 3D** | **Figure 3E** | **Figure 3F** |
|---|---|---|---|---|
| 1 mmol/L Pi vesus 2 mmol/L Pi | <0.0001 | <0.0001 | <0.0001 | <0.0001 |
| 1 mmol/L Pi vesus 2 mmol/L Pi + Mg | 0.0014 | <0.0001 | <0.0001 | <0.0001 |
| 1 mmol/L Pi vesus 2 mmol/L Pi + PPi | 0.8959 | 0.987 | 0.7854 | 0.9455 |
| 1 mmol/L Pi vesus 2 mmol/L Pi + PFA | 0.8483 | 0.9662 | 0.9811 | 0.9727 |
| 2 mmol/L Pi versus 2 mmol/L Pi + Mg | <0.0001 | 0.9284 | <0.0001 | 0.9984 |
| 2 mmol/L Pi versus 2 mmol/L Pi + PPi | <0.0001 | <0.0001 | <0.0001 | <0.0001 |
| 2 mmol/L Pi versus 2 mmol/L Pi + PFA | <0.0001 | <0.0001 | <0.0001 | <0.0001 |
| 2 mmol/L Pi + Mg versus 2 mmol/L Pi +PPi | 0.021 | <0.0001 | <0.0001 | <0.0001 |
| 2 mmol/L Pi + Mg versus 2 mmol/L Pi +PFA | 0.0285 | <0.0001 | <0.0001 | <0.0001 |
| 2 mmol/L Pi + PPi versus 2 mmol/L Pi +PFA | >0.9999 | 0.9999 | 0.9767 | >0.9999 |
| | One-way ANOVA and Tukey´s post hoc test | | | |

**Appendix Table S6**. The exact P values for Figure 4

| | **Figure 4A** | **Figure 4B** | **Figure 4D** | **Figure 4C** |
|---|---|---|---|---|
| wild-type versus untreated Lmna$^{G609G/+}$ | ---- | ---- | ---- | <0.0001 |
| wild-type versus treated Lmna$^{G609G/+}$ | ---- | ---- | ---- | 0.0196 |
| untreated Lmna$^{G609G/+}$ versus treated Lmna$^{G609G/+}$ | <0.0001 | 0,0018 | 0.0034 | <0.0001 |
| | Unpaired t test | | | One-way ANOVA and Tukey´s post hoc test |

**Appendix Table S7**. The exact P values for Figure EV3

| | Figure EV3A | Figure EV3B | Figure EV3C | Figure EV3D | Figure EV3E |
|---|---|---|---|---|---|
| wild-type versus untreated Lmna$^{G609G/+}$ | <0.0001 | <0.0001 | <0.0001 | <0.0001 | <0.0001 |
| wild-type versus treated Lmna$^{G609G/+}$ | 0.0002 | <0.0001 | <0.0001 | <0.0001 | <0.0001 |
| untreated Lmna$^{G609G/+}$ versus treated Lmna$^{G609G/+}$ | 0.0334 | 0.3205 | <0.0001 | <0.0001 | 0.2132 |
| | One-way ANOVA and Tukey´s post hoc test | | | | |

**Appendix Table S8**. The exact P values for Figure 5

| | Figure 5A | Figure 5B | Figure 5C |
|---|---|---|---|
| wild-type versus untreated Lmna$^{G609G/+}$ | <0.0001 | <0.0001 | <0.0001 |
| wild-type versus treated Lmna$^{G609G/+}$ | 0.0034 | 0.1039 | 0.3034 |
| untreated Lmna$^{G609G/+}$ versus treated Lmna$^{G609G/+}$ | 0.001 | <0.0001 | 0.0064 |
| | One-way ANOVA and Tukey´s post hoc test | | |

| | Figure 5D Complex I | Complex II | Complex III | Complex IV | Complex V |
|---|---|---|---|---|---|
| wild-type versus untreated Lmna$^{G609G/+}$ | <0.0001 | 0.374 | 0.0002 | <0.0001 | <0.0001 |
| wild-type versus treated Lmna$^{G609G/+}$ | 0.0049 | 0.4839 | 0.1103 | <0.0001 | 0.0011 |
| untreated Lmna$^{G609G/+}$ versus treated Lmna$^{G609G/+}$ | <0.0001 | 0.0108 | 0.0402 | <0.0001 | <0.0001 |
| | One-way ANOVA and Tukey´s post hoc test | | | | |

**Appendix Table S9**. The exact P values for Figure EV4

| | Figure EV4A | | Figure EV4B | |
|---|---|---|---|---|
| | 0.1 mmol/L | 1 mmol/L | | |
| wild-type versus untreated Lmna$^{G609G/+}$ | <0.0001 | <0.0001 | <0.0001 | <0.0001 |
| wild-type versus treated Lmna$^{G609G/+}$ | 0.0024 | <0.0001 | 0.1326 | 0.0005 |
| untreated Lmna$^{G609G/+}$ versus treated Lmna$^{G609G/+}$ | 0.0068 | 0.0001 | <0.0001 | <0.0001 |
| | One-way ANOVA and Tukey´s post hoc test | | | |
