# Supplementary material for: Dietary magnesium supplementation improves lifespan in a mouse model of progeria
Source: EMBO Mol Med. 2020 Aug 16;12(10):e12423. doi: 10.15252/emmm.202012423 (PMC7539193; doi:10.15252/emmm.202012423)
Supplement: Supplementary file 2 — Expanded View Figures PDF [file EMMM-12-e12423-s002.pdf]

Expanded View Figures

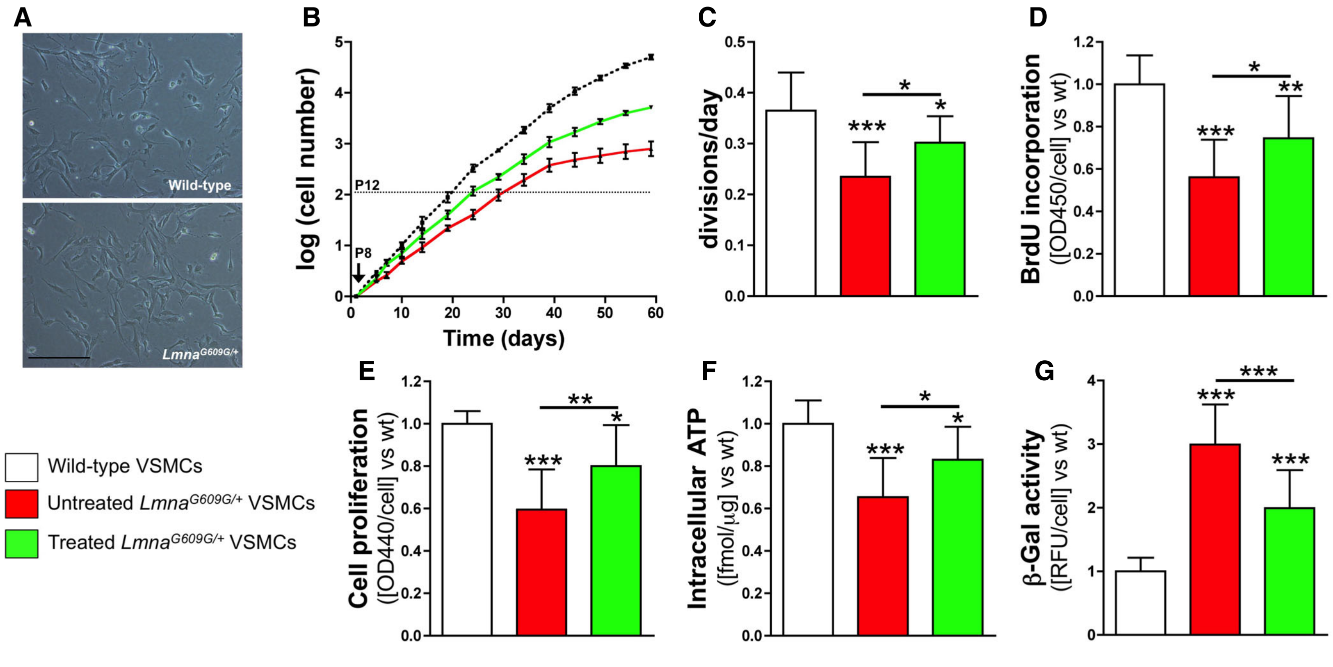

Figure EV1. Magnesium improves *Lmna*$^{G609G/+}$ VSMC viability.

VSMCs were incubated in MEM containing 10% FBS and 0.8 mM magnesium (wild-type and untreated *Lmna*$^{G609G/+}$ VSMCs) or 1.8 mM magnesium (treated *Lmna*$^{G609G/+}$ VSMCs) from passage 1 to passage 8 (P8).

A Representative microscopy images (10x; scale bar: 100 μm) of wild-type and *Lmna*$^{G609G/+}$ VSMCs at passage 10.
B Number of replicative cells at the indicated times. Cell count begins at passage 8 and ends after 60 days.
C Mean number of divisions per day over the first 30 days.
D Replicative incorporation of 5-bromodeoxyuridine (BrdU) into DNA.
E Cell viability measured using the cleavage of tetrazolium salt by cellular mitochondria dehydrogenases.
F Intracellular ATP content.
G β-galactosidase (β-Gal) activity.

Data information: Results are presented as the mean ± SD of three independent experiments (four wells *per* experiment). One-way ANOVA and Tukey's multiple comparisons *post hoc* test were used for statistical analysis. $^{*}P < 0.05$; $^{**}P < 0.01$; $^{***}P < 0.001$.
Source data are available online for this figure.

© 2020 The Author

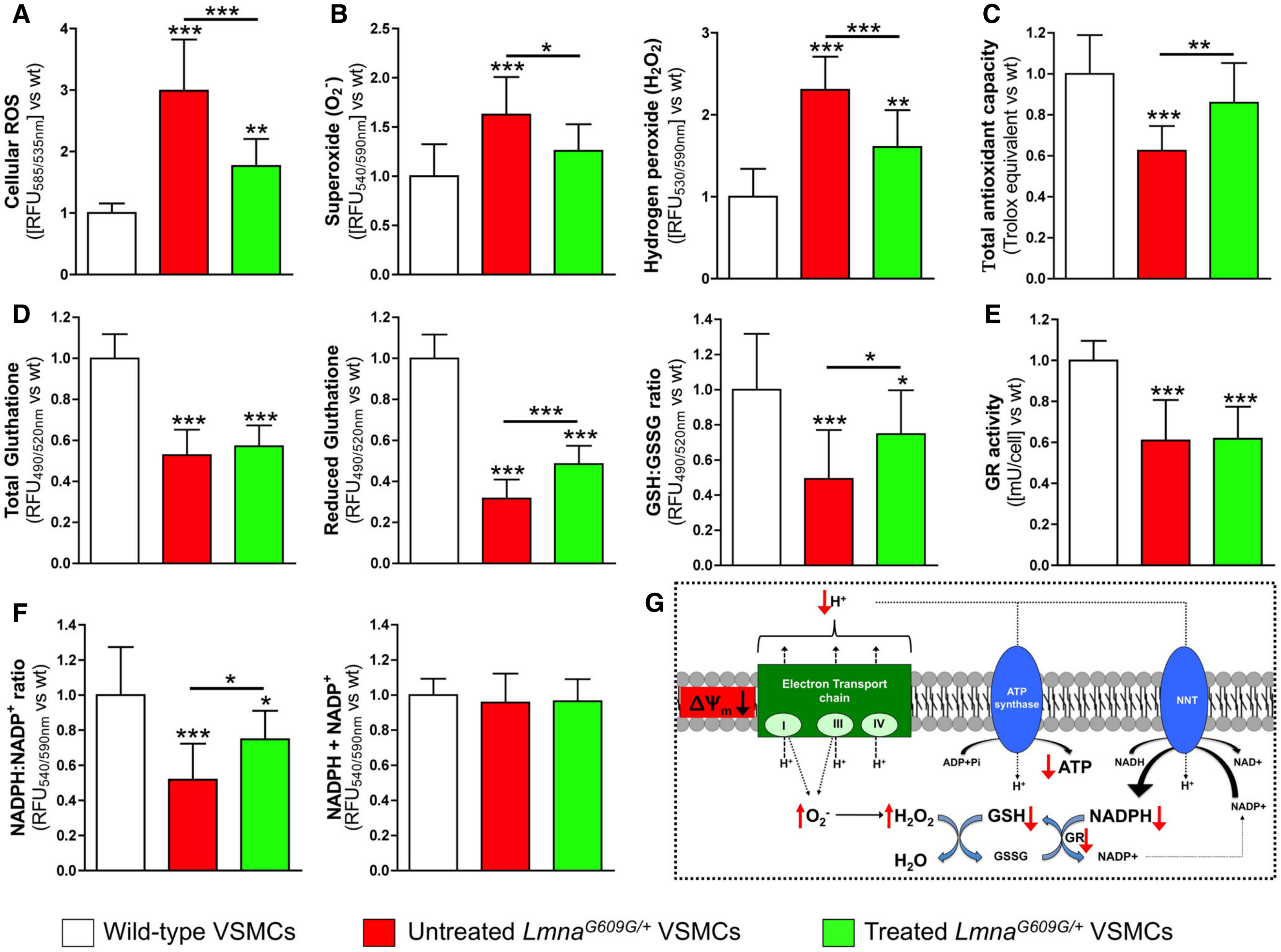

Figure EV2. Magnesium ameliorates oxidative stress in *Lmna*$^{G609G/+}$ VSMCs.

A, B (A) Reactive oxygen species, and (B) superoxide and hydrogen peroxide radicals generated by the indicated VSMC types.

C, D (C) Total antioxidant capacity and (D) total glutathione (which includes reduced -GSH- and oxidized -GSSG- glutathione), reduced glutathione (GHS), and the ratio of reduced and oxidized glutathione (GSSG) in the indicated cell types.

E, F (E) Glutathione reductase (GR) activity, and (F) NADPH:NADP$^+$ ratio and total NADPH (NADPH + NADP$^+$) in the indicated VSMC types.

G The boxed scheme shows the NADPH-coupled glutathione redox systems, H$^+$-coupled ATP synthesis by mitochondrial ATP synthase, and H$^+$-coupled synthesis of NADPH by mitochondrial NADPH transhydrogenase (NNT). $\Delta\Psi_m$: mitochondrial membrane potential.

Data information: Results are presented as the mean ± SD of three independent experiments (four wells per experiment). One-way ANOVA and Tukey's multiple comparisons *post hoc* test were used for statistical analysis. *$P < 0.05$; **$P < 0.01$; ***$P < 0.001$.
Source data are available online for this figure.

© 2020 The Author

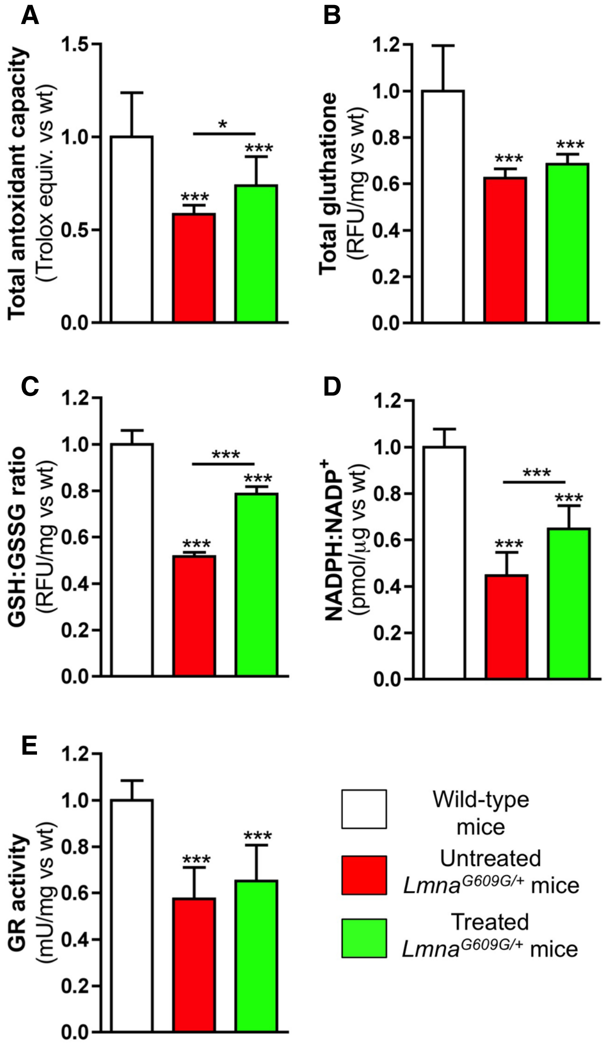

Figure EV3. Magnesium improves the NADPH-coupled glutathione redox status in *Lmna*$^{G609G/+}$ mice.

Liver homogenates were obtained from 34-week-old wild-type, untreated, or treated *Lmna*$^{G609G/+}$ mice.

A–C (A) Total antioxidant capacity, (B) total glutathione (which includes reduced and oxidized glutathione), and (C) the ratio of reduced (GSH) and oxidized (GSSG) in the indicated experimental mouse groups.

D, E (D) The NADPH:NADP$^+$ ratio and (E) glutathione reductase (GR) activity.

Data information: Results are presented as mean ± SD (n = 16). One-way ANOVA and Tukey's multiple comparisons *post hoc* test were used for statistical analysis. *P < 0.05; ***P < 0.001.
Source data are available online for this figure.

 © 2020 The Author

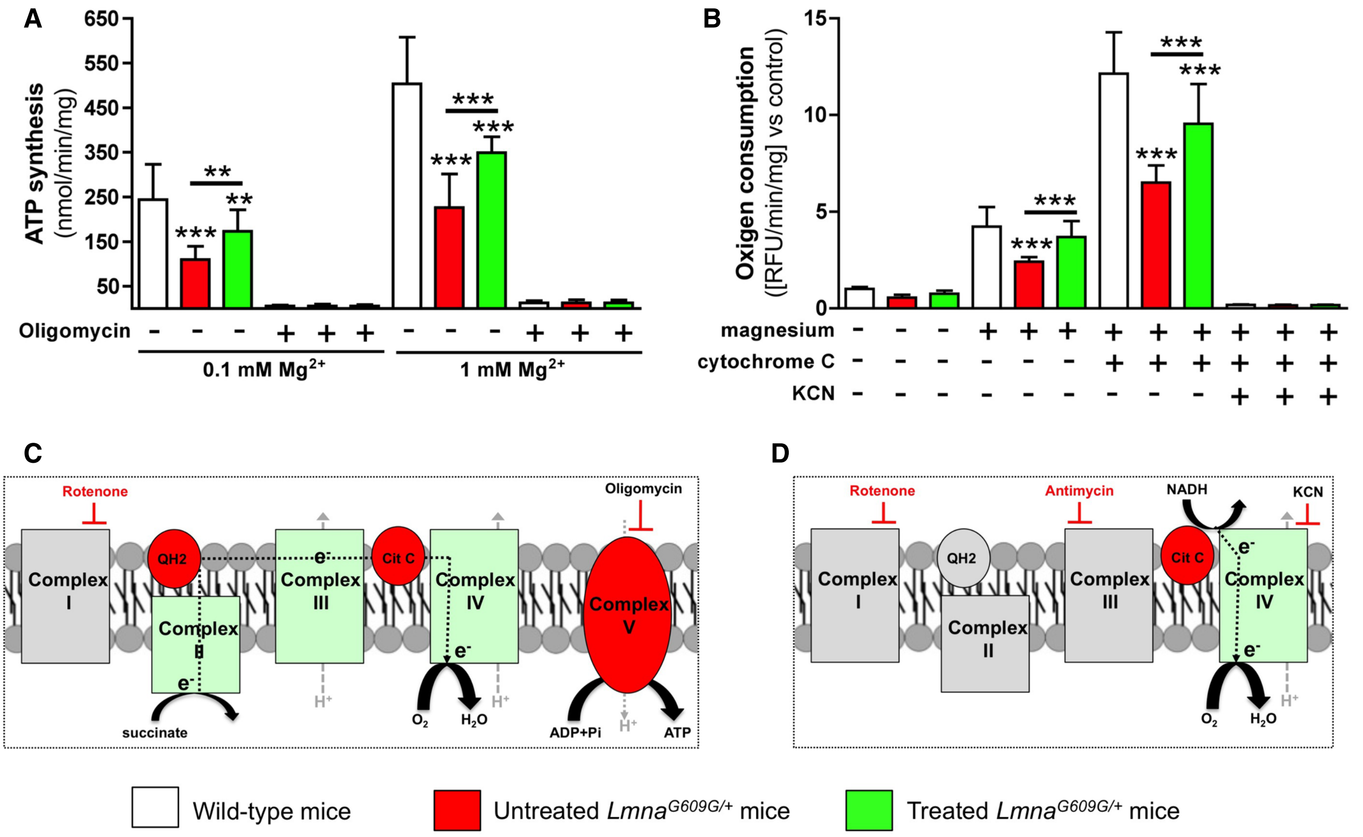

Figure EV4. Magnesium increases the activity of mitochondrial ATP synthase and extramitochondrial NADH oxidation.

A ATP synthase activity was measured in mitochondria isolated from the livers of 34-week-old wild-type, untreated, or treated *Lmna*$^{G609G/+}$ mice in media containing 0.1 mM magnesium or 1 mM magnesium. The isolated mitochondria were also incubated in the absence or presence of oligomycin (10 µg/ml). The incubation media contain 5 mM succinate and 2 µM rotenone. The isolated mitochondria were also incubated in the absence or presence of oligomycin (10 µM).

B Extramitochondrial NADH oxidation was measured in mitochondrial isolates from the livers of wild-type, untreated, or treated *Lmna*$^{G609G/+}$ mice in media containing rotenone (2 µM), antimycin A (10 µM), and NADH (1 mmo/L). The isolated mitochondria were also incubated in the absence or presence of $MgCl_2$ (5 mM), cytochrome C (10 µM), or KCN (1 mM).

C, D The boxed scheme shows the five mitochondrial complexes involved in the electron transport chain and their known inhibitors used in the experiment.

Data information: Results are presented as mean ± SD (n = 16). One-way ANOVA and Tukey's multiple comparisons *post hoc* test were used for statistical analysis. $^{**}P < 0.01$; $^{***}P < 0.001$.

Source data are available online for this figure.
