# Supplementary material for: Dietary magnesium supplementation improves lifespan in a mouse model of progeria
Source: EMBO Mol Med. 2020 Aug 16;12(10):e12423. doi: 10.15252/emmm.202012423 (PMC7539193; doi:10.15252/emmm.202012423)
Supplement: Supplementary file 3 — Table EV1 [file EMMM-12-e12423-s003.pdf]

Table EV1. Magnesium levels in mice plasma. Results are presented as the mean ± S.D (n=20). Statistical analyses were performed using Student's *t*-test.

		**Mean± S.D (mmol/L)**	***p***
8 weeks	wild-type	0,97 ± 0.09	
	Lmna$^{G609G/+}$	0,96 ± 0.12	*0.685*
21 weeks	wild-type	1,00 ± 0.09	
	Lmna$^{G609G/+}$	0,95 ± 0.06	*0.046*
34 weeks	wild-type	1,01 ± 0.08	
	Lmna$^{G609G/+}$	0,96 ± 0.05	*0.017*
