# Supplementary material for: Dietary magnesium supplementation improves lifespan in a mouse model of progeria
Source: EMBO Mol Med. 2020 Aug 16;12(10):e12423. doi: 10.15252/emmm.202012423 (PMC7539193; doi:10.15252/emmm.202012423)
Supplement: Supplementary file 4 — Source Data for Expanded View [file EMMM-12-e12423-s009.zip › Data_source_FigureEV4.pdf]

Figure 9A

0.1 mmol/L Mg^{2+}			1 mmol/L Mg^{2+}		
wild-type	untreated *Lmna*$^{G609G/+}$	treated *Lmna*$^{G609G/+}$	wild-type	untreated *Lmna*$^{G609G/+}$	treated *Lmna*$^{G609G/+}$
225,44	125,64	177,05	545,81	210,78	338,76
239,53	127,77	173,32	559,80	219,31	325,71
127,21	85,08	254,06	612,04	279,14	334,05
131,16	88,51	245,60	668,97	281,55	338,97
264,45	136,74	228,83	660,38	224,07	333,09
270,53	139,22	233,33	657,84	214,89	323,38
199,71	83,70	181,01	477,22	183,86	380,09
223,52	95,38	188,93	513,58	200,31	383,76
355,99	83,64	160,80	445,42	184,87	366,03
365,81	87,65	160,21	447,74	183,59	347,34
143,51	117,47	141,01	414,97	148,66	303,42
144,56	119,42	139,01	466,00	149,09	329,17
308,57	71,54	156,21	342,04	383,43	302,14
328,21	75,78	139,89	368,25	401,36	346,61
274,96	158,05	94,33	409,40	170,18	413,29
302,14	162,18	99,07	468,92	184,78	423,25

Figure 9B

wild-type	untreated *Lmna*$^{G609G/+}$	treated *Lmna*$^{G609G/+}$	wild-type	untreated *Lmna*$^{G609G/+}$	treated *Lmna*$^{G609G/+}$	wild-type	untreated *Lmna*$^{G609G/+}$	treated *Lmna*$^{G609G/+}$	
0,9976	0,6634	0,9283	2,9199	2,4273	4,4777	13,9570	7,1392	6,9202	
0,9577	0,6781	0,9150	2,8786	2,3552	4,5971	14,3518	7,2057	6,8449	
0,8539	0,4100	0,5027	3,3882	2,1197	2,9553	10,0775	4,8918	8,0179	
0,9145	0,4242	0,5101	3,3859	2,2607	3,0563	10,3759	4,9836	7,9968	
0,8246	0,3870	0,7295	4,8459	2,0311	2,8098	9,6275	6,7260	11,1523	
0,9384	0,3820	0,7378	4,9905	2,3047	2,7671	9,4898	6,7696	11,3318	
1,1156	0,6887	0,7915	5,0158	2,6867	4,6141	14,2187	6,9257	11,3400	
1,1267	0,6942	0,8085	5,1673	2,6876	4,6108	14,1452	6,9991	11,4984	
1,0647	0,6900	0,9384	3,1747	2,5531	4,5576	14,0212	7,1323	7,2204	
1,0284	0,7075	0,9375	3,1265	2,4870	3,0150	14,4895	7,2080	7,1497	
0,9219	0,4430	0,5431	3,6660	2,2529	3,1371	10,4815	5,0984	8,3558	
0,9848	0,4609	0,5523	3,6614	2,3933	3,2399	10,7616	5,1834	8,3264	
0,8865	0,4201	0,7800	5,2155	2,1587	2,9920	10,0408	6,8729	11,5278	
1,0087	0,4137	0,7883	5,3486	2,4333	2,9475	9,8938	6,8981	11,6779	
1,1863	0,7139	0,8360	5,3762	2,7955	4,6692	14,2783	7,0221	11,6320	
1,1896	0,7153	0,8498	5,4864	2,7836	4,6430	14,0947	7,0611	11,7574	
-	-	-	+	+	+	+	+	+	magnesium
-	-	-	-	-	-	+	+	+	cytochrome C
-	-	-	-	-	-	-	-	-	KCN
