# Supplementary material for: Dietary magnesium supplementation improves lifespan in a mouse model of progeria
Source: EMBO Mol Med. 2020 Aug 16;12(10):e12423. doi: 10.15252/emmm.202012423 (PMC7539193; doi:10.15252/emmm.202012423)
Supplement: Supplementary file 4 — Source Data for Expanded View [file EMMM-12-e12423-s009.zip › Data_source_FigureEV1.pdf]

divisions/day			
wild-type	untreated *Lmna*$^{G609G/+}$	treated *Lmna*$^{G609G/+}$	VSMCs
0,3322	0,2799	0,3163	
0,3814	0,2757	0,3414	
0,3064	0,2701	0,2504	
0,3063	0,2479	0,2427	
0,3159	0,2708	0,2324	
0,4858	0,2253	0,2925	
0,3839	0,2689	0,4401	
0,2860	0,3466	0,2861	
0,4744	0,1735	0,3509	
0,5014	0,3181	0,3048	
0,2738	0,1568	0,2362	
0,3844	0,1585	0,2810	
0,4129	0,2539	0,2854	
0,4380	0,1396	0,3429	
0,3007	0,1811	0,2637	
0,3190	0,1812	0,2863	
0,3965	0,3413	0,3256	
0,2692	0,1392	0,3523	

BrdU Incorporation			
wild-type	untreated *Lmna*$^{G609G/+}$	treated *Lmna*$^{G609G/+}$	VSMCs
1,0449	0,8110	0,8890	
1,0264	0,2916	0,8228	
1,0056	0,7375	0,5376	
0,9231	0,6803	0,6748	
1,0108	0,3849	0,9844	
0,8181	0,6971	0,9112	
1,2559	0,3516	0,9996	
0,9152	0,4677	0,9225	
1,1924	0,4676	0,6401	
0,9018	0,4551	0,4442	
0,8114	0,7570	0,4871	
1,0944	0,6327	0,6330	

Cell proliferation		
wild-type	untreated *Lmna*$^{G609G/+}$	treated *Lmna*$^{G609G/+}$
0,9870	0,8652	0,9114
1,0282	0,5253	1,0917
0,9767	0,6808	0,9307
1,0081	0,7780	1,1037
1,1073	0,6038	0,6520
1,0146	0,8164	0,7896
0,9475	0,7496	0,8384
0,9306	0,6159	0,5730
1,0969	0,3045	0,8685
0,9660	0,4425	0,7112
0,9154	0,3899	0,5010
1,0217	0,3641	0,6311

VSMCs

Intracellular ATP		
wild-type	untreated *Lmna*$^{G609G/+}$	treated *Lmna*$^{G609G/+}$
1,0724	0,8765	0,8346
1,1101	0,4810	0,9997
0,8943	0,6234	0,8523
0,9231	0,7124	1,0106
1,1554	0,9259	0,8404
0,9977	0,3378	0,9093
0,9318	0,7371	0,9573
0,9152	0,7772	0,5635
0,7955	0,5925	0,9587
1,0663	0,7316	0,7850
1,0104	0,3612	0,5530
1,1278	0,6787	0,6966

VSMCs

b-gal activity			
wild-type	untreated *Lmna*$^{G609G/+}$	treated *Lmna*$^{G609G/+}$	VSMCs
0,9016	3,1420	2,3063	
0,6184	3,0365	2,2588	
1,4933	2,4987	1,2983	
0,9868	1,6357	1,3938	
1,0275	3,3322	2,3454	
1,0310	3,0441	1,9784	
1,1242	2,4524	1,0806	
0,8174	2,9229	1,2864	
0,8887	3,1124	3,0276	
0,8831	4,1032	2,2840	
1,1671	2,8898	2,5054	
1,0611	3,7523	2,1568	
