# Supplementary material for: Dietary magnesium supplementation improves lifespan in a mouse model of progeria
Source: EMBO Mol Med. 2020 Aug 16;12(10):e12423. doi: 10.15252/emmm.202012423 (PMC7539193; doi:10.15252/emmm.202012423)
Supplement: Supplementary file 4 — Source Data for Expanded View [file EMMM-12-e12423-s009.zip › Data_source_FigureEV2.pdf]

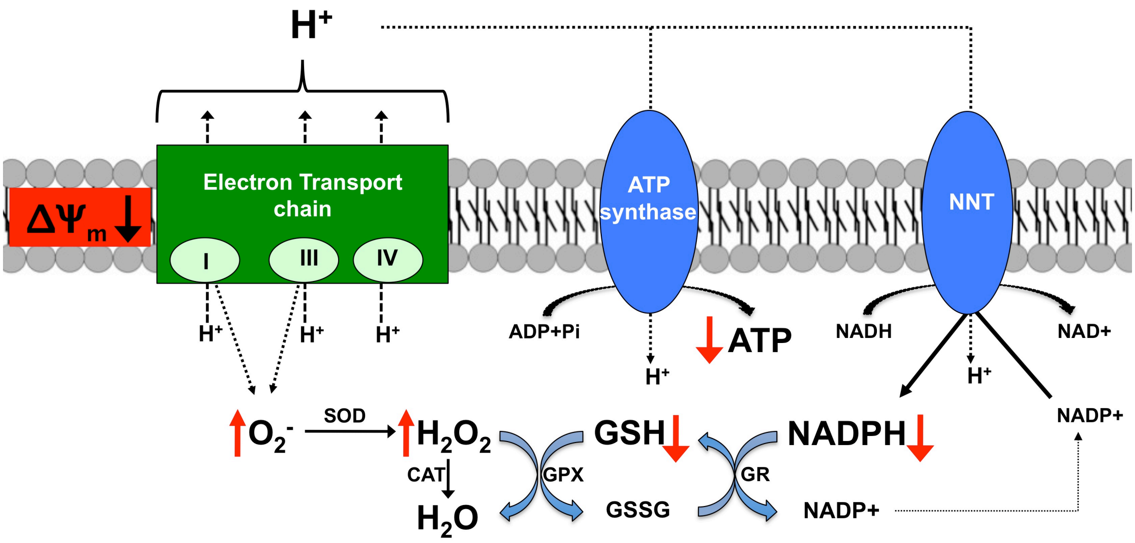
H+
ΔΨm
Electron Transport chain
I
III
IV
H+
H+
H+
ATP synthase
ADP+Pi
ATP
H+
NNT
NADH
NAD+
H+
NADP+
O2-
SOD
H2O2
CAT
H2O
GSH
GPX
GSSG
GR
NADPH
NADP+

Cellular ROS			
**wild-type**	**untreated *Lmna*$^{G609G/+}$**	**treated *Lmna*$^{G609G/+}$**	**VSMCs**
**1,0320**	**2,3523**	**1,8282**	
**0,9929**	**2,5962**	**1,5884**	
**1,0091**	**2,0802**	**1,7299**	
**0,9660**	**2,5706**	**1,9630**	
**1,2174**	**4,4910**	**1,7678**	
**0,9309**	**2,9422**	**1,2992**	
**0,7632**	**4,2905**	**1,1621**	
**1,0885**	**2,7990**	**2,2322**	
**0,8301**	**3,6589**	**1,6267**	
**1,1664**	**3,3226**	**2,5903**	
**0,7847**	**3,0125**	**1,1688**	
**1,2188**	**1,7269**	**2,2063**	

Hydrogen peroxide			
**wild-type**	**untreated *Lmna*$^{G609G/+}$**	**treated *Lmna*$^{G609G/+}$**	**VSMCs**
**1,1563**	**2,5257**	**2,0815**	
**1,2176**	**1,7946**	**1,8761**	
**0,6283**	**2,0011**	**1,6897**	
**0,9978**	**2,4018**	**2,0379**	
**1,4150**	**2,8946**	**0,8885**	
**1,3383**	**2,3222**	**1,0138**	
**0,4901**	**2,4325**	**1,3149**	
**0,7566**	**2,3858**	**2,1548**	
**1,4712**	**2,7873**	**1,1447**	
**0,9369**	**2,5298**	**1,6487**	
**0,5222**	**1,4783**	**1,3942**	
**1,0697**	**2,1120**	**2,0553**	

Total Gluthatione			Reduced Gluthatione		
wild-type	untreated *Lmna*$^{G609G/+}$	treated *Lmna*$^{G609G/+}$	wild-type	untreated *Lmna*$^{G609G/+}$	treated *Lmna*$^{G609G/+}$
**0,8290**	**0,4629**	**0,6088**	**1,0725**	**0,3491**	**0,6105**
**1,0692**	**0,4545**	**0,5753**	**1,1094**	**0,4488**	**0,5909**
**1,0219**	**0,4455**	**0,6446**	**0,8948**	**0,2251**	**0,5895**
**1,0799**	**0,4090**	**0,5549**	**0,9233**	**0,4125**	**0,5777**
**1,1366**	**0,4867**	**0,4734**	**1,1551**	**0,3925**	**0,4117**
**0,9023**	**0,3941**	**0,4672**	**0,9975**	**0,1235**	**0,4458**
**0,8346**	**0,6047**	**0,5744**	**0,9322**	**0,4063**	**0,3898**
**1,1265**	**0,4405**	**0,5637**	**0,9152**	**0,4063**	**0,3547**
**0,9165**	**0,8207**	**0,8128**	**0,8674**	**0,2802**	**0,3844**
**1,0740**	**0,6206**	**0,5811**	**1,1626**	**0,2178**	**0,4874**
**0,9152**	**0,5585**	**0,6013**	**1,1176**	**0,2271**	**0,4312**
**1,0943**	**0,7533**	**0,7087**	**0,8524**	**0,3151**	**0,4618**
**0,9197**	**0,4358**	**0,3970**	**1,1147**	**0,3826**	**0,4417**
**1,1524**	**0,4634**	**0,5196**	**0,8121**	**0,3545**	**0,5601**
**0,8279**	**0,6100**	**0,6103**	**1,0708**	**0,2312**	**0,6052**
**1,1000**	**0,4992**	**0,4578**	**1,0024**	**0,2910**	**0,4033**

NADPH+NADP			NADPH:NADP ratio		
wild-type	untreated *Lmna*$^{G609G/+}$	treated *Lmna*$^{G609G/+}$	wild-type	untreated *Lmna*$^{G609G/+}$	treated *Lmna*$^{G609G/+}$
**1,1728**	**0,7809**	**1,1018**	**0,9900**	**0,5890**	**0,7812**
**1,0544**	**0,7232**	**1,0449**	**1,5056**	**0,6830**	**0,6792**
**0,9146**	**1,1622**	**1,1817**	**0,6472**	**0,2491**	**0,7816**
**0,8582**	**1,1542**	**0,9425**	**0,8571**	**0,3458**	**0,5956**
**0,9575**	**0,8007**	**0,7644**	**1,1884**	**0,9090**	**1,0507**
**1,0672**	**0,8844**	**0,8993**	**1,1571**	**0,6621**	**0,6527**
**0,9638**	**0,9515**	**0,8747**	**0,8788**	**0,6977**	**0,8806**
**1,0115**	**0,9531**	**0,9198**	**0,7756**	**0,6359**	**0,8983**
**1,0330**	**1,1318**	**0,9094**	**0,9398**	**0,3701**	**0,6975**
**1,0643**	**1,2006**	**1,1179**	**1,1692**	**0,2691**	**0,5572**
**1,0395**	**0,8653**	**0,9877**	**0,5851**	**0,3997**	**0,5093**
**0,8632**	**0,8779**	**0,8321**	**1,3059**	**0,3991**	**0,8967**
