# Supplementary material for: Dietary magnesium supplementation improves lifespan in a mouse model of progeria
Source: EMBO Mol Med. 2020 Aug 16;12(10):e12423. doi: 10.15252/emmm.202012423 (PMC7539193; doi:10.15252/emmm.202012423)
Supplement: Supplementary file 4 — Source Data for Expanded View [file EMMM-12-e12423-s009.zip › Data_source_FigureEV3.pdf]

Total antioxidant capacity		
wild-type	untreated *Lmna*$^{G609G/+}$	treated *Lmna*$^{G609G/+}$
0,970	0,616	0,677
0,768	0,610	0,665
0,811	0,566	0,747
0,811	0,552	0,723
1,324	0,608	0,941
1,334	0,613	0,941
0,987	0,512	0,511
0,963	0,495	0,547
0,668	0,653	0,718
0,815	0,642	0,704
0,851	0,594	0,794
0,856	0,578	0,768
1,395	0,624	0,979
1,403	0,627	0,975
1,034	0,538	0,535
1,008	0,521	0,573

Liver

Total gluthatione			GSH:GSSG ratio		
wild-type	untreated *Lmna*$^{G609G/+}$	treated *Lmna*$^{G609G/+}$	wild-type	untreated *Lmna*$^{G609G/+}$	treated *Lmna*$^{G609G/+}$
1,139	0,620	0,676	0,946	0,501	0,759
1,166	0,630	0,675	0,947	0,505	0,765
0,721	0,641	0,751	0,963	0,501	0,751
0,698	0,659	0,752	0,966	0,502	0,752
1,171	0,580	0,672	0,950	0,507	0,768
1,166	0,579	0,696	0,951	0,509	0,764
0,992	0,666	0,640	0,969	0,500	0,772
1,069	0,682	0,689	0,956	0,496	0,759
1,110	0,602	0,654	1,007	0,535	0,821
1,141	0,612	0,657	1,000	0,533	0,816
0,683	0,624	0,742	1,125	0,530	0,767
0,663	0,648	0,748	1,119	0,519	0,761
1,148	0,560	0,649	0,993	0,550	0,832
1,146	0,558	0,675	0,991	0,550	0,815
0,957	0,657	0,618	1,063	0,513	0,839
1,030	0,674	0,666	1,053	0,508	0,822

GR activity			NADPH:NADP ratio		
wild-type	untreated *Lmna*$^{G609G/+}$	treated *Lmna*$^{G609G/+}$	wild-type	untreated *Lmna*$^{G609G/+}$	treated *Lmna*$^{G609G/+}$
1,036	0,592	0,672	0,922	0,504	0,863
1,032	0,619	0,688	0,931	0,525	0,886
1,071	0,520	0,613	1,065	0,671	0,708
0,993	0,508	0,573	1,082	0,673	0,700
0,860	0,506	0,758	1,134	0,398	0,584
0,874	0,512	0,754	1,107	0,400	0,629
0,945	0,426	0,605	0,873	0,404	0,616
0,937	0,700	0,404	0,886	0,376	0,608
1,122	0,821	0,704	0,983	0,411	0,649
1,124	0,753	0,721	0,992	0,419	0,657
1,116	0,576	0,658	1,004	0,449	0,612
1,036	0,564	0,613	1,020	0,451	0,612
0,920	0,549	0,809	1,058	0,365	0,545
0,932	0,556	0,801	1,040	0,365	0,555
1,001	0,255	0,233	0,949	0,368	0,562
1,001	0,735	0,825	0,953	0,362	0,569
