# Supplementary material for: Dietary magnesium supplementation improves lifespan in a mouse model of progeria
Source: EMBO Mol Med. 2020 Aug 16;12(10):e12423. doi: 10.15252/emmm.202012423 (PMC7539193; doi:10.15252/emmm.202012423)
Supplement: Supplementary file 6 — Source Data for Figure 1 [file EMMM-12-e12423-s004.pdf]

Mitochondrial ATP		
**wild-type**	**untreated *Lmna*$^{G609G/+}$**	**treated *Lmna*$^{G609G/+}$**
1,1957	0,6108	0,6650
0,9500	0,5621	0,9140
0,8077	0,4972	0,8610
1,0465	0,4205	0,8045
1,0747	0,5683	0,5056
0,9643	0,7102	0,7581
0,9349	0,4154	0,7926
1,0262	0,5353	0,7309
1,1912	1,0582	0,8677
0,9548	0,4863	0,8839
0,8532	0,5644	0,8582
1,0009	0,8970	0,9458

VSMCs

Mitochondrial MP		
**wild-type**	**untreated *Lmna*$^{G609G/+}$**	**treated *Lmna*$^{G609G/+}$**
0,9611	0,6018	0,8662
1,1199	0,5895	0,6787
0,9188	0,4636	1,0666
1,0002	0,5664	0,6414
1,0324	0,7052	1,0906
0,9728	0,8359	0,8116
0,9017	0,7607	0,6740
1,0931	0,6431	0,4463
0,9057	0,4793	1,0260
1,0586	0,9119	1,0816
1,0773	0,4923	0,7511
0,9584	0,5615	0,8791

VSMCs
