# Supplementary material for: Dietary magnesium supplementation improves lifespan in a mouse model of progeria
Source: EMBO Mol Med. 2020 Aug 16;12(10):e12423. doi: 10.15252/emmm.202012423 (PMC7539193; doi:10.15252/emmm.202012423)
Supplement: Supplementary file 7 — Source Data for Figure 2 [file EMMM-12-e12423-s005.pdf]

Intracellular Lactate			
wild-type	untreated *Lmna*$^{G609G/+}$	treated *Lmna*$^{G609G/+}$	**VSMCs**
1,178	1,911	1,316	
0,948	1,776	1,776	
0,822	1,615	1,617	
1,052	1,394	1,520	
1,052	1,796	1,369	
0,955	2,218	1,434	
0,949	1,362	1,490	
1,044	1,738	1,386	
1,170	3,095	1,691	
0,952	1,550	1,732	
0,868	1,825	1,605	
1,010	2,826	1,769	

Acidification			
wild-type	untreated *Lmna*$^{G609G/+}$	treated *Lmna*$^{G609G/+}$	**VSMCs**
0,690	1,544	1,343	
1,154	2,302	2,011	
1,134	2,318	1,961	
1,022	1,984	1,587	
1,011	2,030	1,788	
0,888	1,929	1,679	
1,019	2,067	1,680	
1,081	2,089	1,780	
1,010	1,963	1,843	
0,862	2,037	1,801	
1,195	2,550	2,060	
0,933	2,033	1,710	
0,902	1,857	1,592	
1,118	2,385	1,997	
0,927	2,089	1,813	
1,053	2,235	1,980	
-	-	-	2-DG

Cytosolic ATP			
**wild-type**	**untreated** ***Lmna***$^{G609G/+}$	**treated** ***Lmna***$^{G609G/+}$	**VSMCs**
**0,800**	**2,285**	**2,582**	
**1,047**	**1,772**	**2,390**	
**1,230**	**2,046**	**1,825**	
**0,923**	**1,882**	**1,960**	
**0,776**	**2,303**	**2,555**	
**1,355**	**2,012**	**2,791**	
**0,904**	**2,019**	**3,062**	
**0,965**	**2,331**	**2,389**	
**1,145**	**1,426**	**1,928**	
**0,911**	**1,785**	**1,846**	
**1,007**	**1,309**	**1,649**	
**0,936**	**1,212**	**2,028**	
**-**	**-**	**-**	**2-DG**
+	+	+	**Oligomicyn**

Mitochondrial calcium-45			Mitochondrial magnesium		
**wild-type**	**untreated** ***Lmna***$^{G609G/+}$	**treated** ***Lmna***$^{G609G/+}$	**wild-type**	**untreated** ***Lmna***$^{G609G/+}$	**treated** ***Lmna***$^{G609G/+}$
**1,067**	**1,442**	**0,950**	**0,733**	**0,698**	**1,157**
**0,870**	**0,805**	**1,231**	**0,992**	**0,847**	**0,597**
**1,132**	**1,231**	**0,735**	**1,512**	**0,618**	**0,869**
**0,931**	**1,500**	**1,260**	**0,762**	**0,345**	**0,795**
**0,994**	**1,483**	**1,248**	**0,969**	**0,373**	**0,805**
**1,024**	**1,811**	**1,010**	**0,810**	**0,839**	**0,884**
**0,946**	**1,559**	**1,439**	**1,458**	**0,664**	**0,772**
**1,037**	**2,029**	**1,202**	**0,763**	**0,477**	**0,987**
**0,927**	**1,869**	**1,116**	**1,091**	**0,762**	**0,838**
**0,963**	**1,432**	**1,306**	**0,700**	**0,597**	**1,069**
**1,065**	**2,197**	**1,469**	**1,486**	**0,680**	**0,738**
**1,045**	**1,195**	**1,718**	**0,723**	**0,890**	**1,194**
