# Supplementary material for: Dietary magnesium supplementation improves lifespan in a mouse model of progeria
Source: EMBO Mol Med. 2020 Aug 16;12(10):e12423. doi: 10.15252/emmm.202012423 (PMC7539193; doi:10.15252/emmm.202012423)
Supplement: Supplementary file 8 — Source Data for Figure 3 [file EMMM-12-e12423-s006.pdf]

Fixed

Untreated Lmna$^{G609G/+}$VSMCs

1	2	2	2	2	mmol/L Pi
		Mg	PPi	PFA	
3,791	85,536	70,977	5,961	2,683	
4,340	66,696	73,470	7,225	3,249	
3,487	45,797	59,190	5,421	4,632	
3,486	59,409	61,091	5,582	4,540	
3,190	52,953	67,668	4,768	5,234	
4,906	74,336	58,433	6,014	6,019	
2,213	58,409	54,571	5,726	5,454	
3,058	52,124	53,600	4,897	4,699	
2,812	46,451	58,893	5,774	5,580	
2,486	54,438	49,022	3,871	5,592	
3,343	46,395	43,539	4,193	6,562	
3,255	55,477	57,048	5,212	6,128	

Fixed

treated Lmna$^{G609G/+}$VSMCs

1	2	2	2	2	mmol/L Pi
		Mg	PPi	PFA	
3,841	71,029	69,427	7,409	3,817	
3,773	90,200	47,985	6,373	4,851	
3,697	53,544	47,016	4,330	4,308	
3,393	57,832	103,171	5,434	3,083	
3,378	49,900	90,674	5,741	5,561	
2,734	75,431	53,612	6,149	4,862	
4,197	66,367	77,723	4,618	6,311	
3,058	52,124	60,262	4,897	6,222	
3,905	50,192	45,538	5,259	9,225	
2,953	70,245	56,230	4,975	6,454	
2,657	41,742	65,002	5,004	10,227	
3,584	61,087	62,817	5,740	8,338	

Living
Untreated Lmna$^{G609G/+}$VSMCs

1	2	2	2	2	mmol/L Pi
		Mg	PPi	PFA	
3,083	40,227	31,855	8,575	3,485	
4,034	56,500	29,486	5,861	4,187	
4,741	33,567	19,266	4,211	5,347	
3,558	39,088	24,182	5,594	3,670	
2,364	31,894	21,112	4,468	4,830	
5,449	42,551	16,049	7,542	2,979	
2,095	34,413	30,184	3,569	4,376	
2,939	32,294	19,979	4,622	3,950	
4,860	51,349	21,791	4,427	6,014	
2,463	30,184	27,813	5,134	4,243	
3,222	34,328	22,074	7,020	5,929	
3,972	43,641	26,999	6,245	5,660	

Living
treated Lmna$^{G609G/+}$VSMCs

1	2	2	2	2	mmol/L Pi
		Mg	PPi	PFA	
3,762	32,686	12,959	3,362	3,331	
4,049	7,696	20,194	4,278	4,233	
2,970	38,264	2,573	9,312	3,760	
3,234	23,594	14,597	5,085	2,691	
1,767	16,043	5,415	8,275	4,448	
4,076	35,009	8,685	0,971	3,889	
3,164	14,655	3,208	4,916	5,049	
2,672	19,493	12,060	4,201	4,977	
1,613	19,468	16,818	7,919	8,510	
3,260	18,947	13,509	3,515	5,954	
4,714	31,519	12,939	3,640	9,434	
3,611	26,342	16,297	5,678	7,692	
