# Supplementary material for: Dietary magnesium supplementation improves lifespan in a mouse model of progeria
Source: EMBO Mol Med. 2020 Aug 16;12(10):e12423. doi: 10.15252/emmm.202012423 (PMC7539193; doi:10.15252/emmm.202012423)
Supplement: Supplementary file 9 — Source Data for Figure 4 [file EMMM-12-e12423-s007.pdf]

Age of death (weeks)	
untreated *Lmna*$^{G609/+}$	treated *Lmna*$^{G609/+}$
**34,4**	**38,4**
**34,6**	**39,7**
**36,3**	**40,1**
**37,3**	**41,0**
**37,4**	**42,3**
**37,9**	**42,3**
**38,0**	**42,7**
**38,1**	**42,9**
**38,3**	**42,9**
**38,4**	**43,1**
**38,9**	**43,3**
**39,7**	**43,7**
**40,6**	**44,0**
**40,7**	**44,3**
**40,9**	**44,4**
**41,3**	**45,4**

mice
