# Supplementary material for: Dietary magnesium supplementation improves lifespan in a mouse model of progeria
Source: EMBO Mol Med. 2020 Aug 16;12(10):e12423. doi: 10.15252/emmm.202012423 (PMC7539193; doi:10.15252/emmm.202012423)
Supplement: Supplementary file 10 — Source Data for Figure 5 [file EMMM-12-e12423-s008.pdf]

Complex I			Complex II		
wild-type	untreated *Lmna*$^{G609G/+}$	treated *Lmna*$^{G609G/+}$	wild-type	untreated *Lmna*$^{G609G/+}$	treated *Lmna*$^{G609G/+}$
0,984	0,464	0,846	1,149	0,987	0,886
0,876	0,644	0,924	1,250	0,702	1,073
1,100	0,653	0,834	1,203	0,998	1,192
1,233	0,808	0,748	0,929	0,994	1,196
1,229	0,722	0,795	1,134	1,174	0,940
0,980	0,752	0,962	0,987	0,796	1,221
1,319	0,571	0,859	0,763	0,681	1,091
1,147	0,503	0,911	0,724	0,947	0,933
0,971	0,550	0,864	1,149	1,008	0,907
0,932	0,623	0,911	1,124	0,717	1,012
1,040	0,597	0,898	1,001	1,030	1,178
1,233	0,589	0,696	0,954	1,044	1,221
0,748	0,597	0,872	1,120	1,181	0,954
0,649	0,636	0,799	1,026	0,792	0,969
0,687	0,589	0,855	0,760	0,666	1,167
0,872	0,576	0,679	0,727	1,066	1,102

Complex III			Complex IV		
wild-type	untreated *Lmna*$^{G609G/+}$	treated *Lmna*$^{G609G/+}$	wild-type	untreated *Lmna*$^{G609G/+}$	treated *Lmna*$^{G609G/+}$
0,783	0,903	0,808	1,005	0,314	0,560
0,778	0,907	0,813	1,027	0,314	0,560
0,950	0,740	0,804	1,251	0,396	0,795
0,976	0,671	0,826	1,237	0,399	0,802
1,217	0,722	1,131	0,759	0,417	0,873
1,256	0,679	1,178	0,738	0,428	0,884
0,778	0,688	0,705	0,838	0,560	0,702
0,757	0,718	0,684	0,841	0,631	0,652
0,950	0,619	1,041	1,030	0,332	0,556
0,955	0,572	1,062	1,059	0,335	0,563
0,972	1,088	0,735	1,280	0,485	0,784
0,950	1,049	0,654	1,273	0,485	0,795
0,959	0,568	0,838	0,952	0,410	0,948
0,933	0,546	0,791	0,930	0,428	0,952
1,397	0,533	0,869	0,881	0,624	0,727
1,393	0,512	0,929	0,898	0,720	0,695

Complex V			Intracellular ATP		
wild-type	untreated *Lmna*$^{G609G/+}$	treated *Lmna*$^{G609G/+}$	wild-type	untreated *Lmna*$^{G609G/+}$	treated *Lmna*$^{G609G/+}$
**0,906**	**0,384**	**0,845**	**0,552**	**0,441**	**0,673**
**0,869**	**0,358**	**1,319**	**0,649**	**0,439**	**0,660**
**0,885**	**0,638**	**0,695**	**0,891**	**0,434**	**0,721**
**0,917**	**0,642**	**0,636**	**0,888**	**0,425**	**0,705**
**0,915**	**0,684**	**0,771**	**1,437**	**0,606**	**1,117**
**0,891**	**0,660**	**0,793**	**1,431**	**0,608**	**1,109**
**1,051**	**0,747**	**0,686**	**1,135**	**0,384**	**0,576**
**1,040**	**0,786**	**0,762**	**1,098**	**0,370**	**0,597**
**1,064**	**0,459**	**1,057**	**1,015**	**0,398**	**0,613**
**1,024**	**0,428**	**1,107**	**0,542**	**0,398**	**0,601**
**1,055**	**0,723**	**0,793**	**0,829**	**0,402**	**0,597**
**1,044**	**0,721**	**0,721**	**0,827**	**0,391**	**0,633**
**1,011**	**0,767**	**0,876**	**1,313**	**0,596**	**1,069**
**0,974**	**0,723**	**0,902**	**1,300**	**0,601**	**1,060**
**1,164**	**0,813**	**0,771**	**1,060**	**0,348**	**0,549**
**1,188**	**0,848**	**0,856**	**1,031**	**0,337**	**0,563**

Mitochondrial calcium			Mitochondrial magnesium		
wild-type	untreated *Lmna*$^{G609G/+}$	treated *Lmna*$^{G609G/+}$	wild-type	untreated *Lmna*$^{G609G/+}$	treated *Lmna*$^{G609G/+}$
**0,775**	**1,593**	**0,885**	**1,049**	**0,798**	**1,037**
**0,800**	**1,603**	**1,650**	**1,122**	**0,769**	**1,043**
**1,016**	**1,547**	**1,349**	**0,741**	**1,102**	**1,514**
**1,169**	**1,957**	**1,075**	**1,045**	**0,526**	**0,902**
**1,205**	**1,905**	**1,591**	**1,127**	**0,825**	**0,823**
**0,981**	**1,284**	**0,895**	**0,985**	**0,601**	**0,648**
**0,736**	**1,260**	**0,939**	**0,999**	**0,591**	**1,097**
**1,331**	**2,177**	**0,971**	**0,899**	**0,516**	**1,032**
**1,318**	**1,641**	**1,614**	**0,849**	**0,483**	**0,720**
**0,910**	**2,463**	**1,464**	**0,981**	**0,578**	**0,957**
**0,639**	**1,966**	**1,554**	**1,261**	**0,608**	**0,765**
**0,709**	**1,791**	**0,661**	**0,929**	**0,806**	**0,566**
**1,069**	**2,754**	**1,346**	**0,750**	**0,512**	**0,594**
**1,075**	**2,119**	**0,908**	**1,241**	**0,724**	**0,592**
**1,245**	**1,781**	**1,365**	**1,203**	**0,558**	**1,243**
**1,021**	**2,403**	**1,694**	**0,817**	**0,601**	**0,758**
